# Detecting QTL and Candidate Genes for Plant Height in Soybean *via* Linkage Analysis and GWAS

**DOI:** 10.3389/fpls.2021.803820

**Published:** 2022-01-21

**Authors:** Jiajing Wang, Bo Hu, Yuliang Jing, Xiping Hu, Yue Guo, Jiankun Chen, Yuxi Liu, Jianhui Hao, Wen-Xia Li, Hailong Ning

**Affiliations:** ^1^Key Laboratory of Soybean Biology, Ministry of Education, Key Laboratory of Soybean Biology and Breeding/Genetics, Ministry of Agriculture, Northeast Agricultural University, Harbin, China; ^2^Suihua Branch of Heilongjiang Academy of Agricultural Science, Suihua, China; ^3^Key Laboratory of Crop Biotechnology Breeding of the Ministry of Agriculture, Beidahuang Kenfeng Seed Co., Ltd., Harbin, China

**Keywords:** soybean, plant height, QTL, QTN, candidate genes

## Abstract

Soybean is an important global crop for edible protein and oil, and plant height is a main breeding goal which is closely related to its plant shape and yield. In this research, a high-density genetic linkage map was constructed by 1996 SNP-bin markers on the basis of a recombinant inbred line population derived from Dongnong L13 × Henong 60. A total of 33 QTL related to plant height were identified, of which five were repeatedly detected in multiple environments. In addition, a 455-germplasm population with 63,306 SNP markers was used for multi-locus association analysis. A total of 62 plant height QTN were detected, of which 26 were detected repeatedly under multiple methods. Two candidate genes, *Glyma.02G133000* and *Glyma.05G240600*, involving in plant height were predicted by pathway analysis in the regions identified by multiple environments and backgrounds, and validated by qRT-PCR. These results enriched the soybean plant height regulatory network and contributed to molecular selection-assisted breeding.

## Introduction

Soybean [*Glycine max* (L.) Merr.] is one of the most important crops in the world as a major source of protein and oil (Feng et al., 2021). Plant height (PH) as the main trait of soybean plant shape is related to soybean yield (Assefa et al., 2019). Low plants result in lower yields, while too high plants are prone to yield reduction due to lodging. Plant height also affects yield through other traits such as number of pods per plant and number of nodes in the main stem (Chang et al., 2018; Li M. W. et al., 2020). PH wass a complex quantitative trait which was controlled by multiple genes and influenced by environmental conditions (Lee et al., 1996).

With the objective to breeding efficiently, QTL mapping for PH were conducted by linkage and genome-wide association analysis (GWAS) analysis. Based on the bi-parent derived populations and linkage analysis (Xu et al., 2017), 238 QTLs associated with plant height had been listed on all 20 chromosomes.^1^ In these researches, most of the linkage maps were constructed by low-throughput molecular markers such as restriction fragment length polymorphism (RFLP), amplified fragment length polymorphism (AFLP), and simple sequence repeat (SSR) markers, which led to low marker density, large genomic region intervals for QTL localization. It was difficult to predict candidate genes and design marker-assisted selection for PH. With the continuous development of molecular markers, high-throughput and high-density single nucleotide polymorphism markers (SNPs) were used as major markers for linkage analysis for mapping QTL (Adewusi et al., 2017; Gomez-Casati et al., 2017; Zhang et al., 2019; Karikari et al., 2020; Tian et al., 2020; Salari et al., 2021; Silva et al., 2021). In order to construct effective linkage intervals to identify QTL, SNP bin maker technology were gradually used in construction of linkage map. Cao et al. (2019) constructed two linkage maps by 3,958 and 2,600 SNP bin markers for two RIL populations, and identified 8 and 12 PH QTL on chromosomes 2,5,6,7,9,10,15,16,17, and 19 explaining 1.8–50.7% of the phenotypic variation, respectively. Wang et al. (2021) constructed a high-density map containing 2225 bin markers and detected 39 PH QTLs on chromosome 5, 6, 7, 9, 10, 12, 15, 16, 18, and 20, and the phenotypic variation explanation (PVE) ranged from 1.11 to 18.99 % based on a recombinant inbred line population of soybean. The second method for detecting QTL was GWAS, which has been extensively studied through recombinant inbred lines and germplasm populations of soybean (Lü et al., 2016; Qi et al., 2020; Song et al., 2020). With the objective of overcoming the shortage of false positives (Sonah et al., 2015), combinations of linkage and association analysis were gradually used in detecting genome regions controlling quantitative traits (Zhang et al., 2019; Song et al., 2020; Li X. et al., 2021). However, few studies combining both methods have been conducted on PH of soybean.

Based on the results of linkage and GWAS, some genes controlling PH formation were gradually mined, such as *GA20ox*, *GA2ox*, *GA3ox* (Fernandez et al., 2009), and *GmDW1* (Li et al., 2018), *Glyma.01G023100*, *Glyma.03G207700*, *Glyma.12G182500*, *Glyma.16G137500*, *Glyma.20G122200*, *Glyma.20G122300* (Jing et al., 2019), and *Glyma.11g145500*,*Glyma.13g139000*, *Glyma.13g339800* and *Glyma.19g006100* (Han et al., 2021). With pleiotropism, some genes controlled simultaneously multiple traits, for example, such as genetic loci *Dw3* and *Ma1* (Higgins et al., 2014), *PH24* (Zhang et al., 2015), and *uqA07-5* (Shen et al., 2018), and genes *GmTOE4a* (Zhao et al., 2015), *GmAP1* (Chen et al., 2020), and *Dt1* (Yue et al., 2021), *GmGIa* and *GmFPA* (Han et al., 2021) control flowering time and PH in soybean. *GmTFL1b* determines PH and growth habit, which a candidate gene for *Dt1* (Liu et al., 2010).

In this research, QTL/QTN localization of soybean plant height was performed *via* linkage analysis of a recombinant inbred lines and GWAS of a 455-germplasm population. In the region of QTL/QTN, candidate genes related to PH formation were predicted and initially validated by qRT-PCR. The objective of this research was to lay foundation for probing genetic basis and molecular assistant selection of PH.

## Materials and Methods

### Plant Populations

Two soybean varieties with large differences in PH, Dongnong L13 obtained from a cross between Heinong 40 and Jiujiao 5640 and Henong 60 obtained from a cross between Beifeng 11 and Hobbit, were used as parents to mate cross in 2008 in Harbin, Heilongjiang Province (E 126.63°, N 45.75°). F_1_ was planted in Yacheng City, Hainan Province (E 109.00°, N 17.50°) in the winter of the same year. After five consecutive generations from 2010 to 2014 by planting in Harbin and Yacheng alternatively, 139 recombinant inbred lines were obtained and a population formed and were used to conduct linkage analysis. Furthermore, a 455-germplasm population, including 4 local soybean varieties, 387 domestic varieties and 44 foreign varieties, was used for GWAS. The variety name was described and published earlier by Li X. et al. (2020).

### Field Trials and Phenotypic Measurement

RIL6013 were planted in eight environments at three locations: Harbin (E 126.63°, N 45.75°), Keshan (E 125.64°, N 48.25°) and Shuangcheng (E 126.92°, N 45.75°). About 455 germplasm resources were planted in Harbin and Shuangyashan (E 131.16°, N 46.64°) in 2018 and 2019, respectively. The detail information for each plant environment was summarized in Supplementary Table 1. The field experiments were conducted in a randomized block in replication design (RBRD). RBRD is a randomized incomplete block design with three replicates used in each environment. With the objective to eliminate the difference of blot among large amounts of lines in a replicate (block), the replicates were divided into multiple sub-blocks which contain about 15 lines. Three ridges were contained in on blot, and the ridges were 3 m in length and 0.67 m in width. The seeds were sowed in single row on the ridges with the plant space set 0.07. The whole experiments were managed as the same as local field production. Ten plants were randomly selected from each blot to determine PH at the maturity stage. The average value of the 10 plants was used as the observation value of the plot, and finally the average value of the three blots was used for QTL and QTN mapping.

### Statistical Analysis of Phenotype Data

Frequency distribution histograms were drawn from the phenotypic data of PH in each environment and descriptive statistics were performed. Analysis of variance (ANOVA) and estimation of generalized heritability were also performed as Li X. et al. (2021). The statistical model for the multi-environment ANOVA for RBRD was showed as follows:


$$x_{eijk}=\mu +E_{e}+R_{j}+B_{k}+RB_{jk}+B_{k}\left(G_{i}\right)+ER_{ej}+EB_{ek}+ERB_{ejk}+EB_{ek}\left(G_{i}\right)+\varepsilon_{eijk}$$

where μ is the grand average, *G*_*i*_ is the *i*th genotype effect, *E*_*e*_ is the *e*th environment effect, *R*_*i*_ is the *j*th replication effect, *B*_*k*_ is the *k*th block in *j*th replicate, *RB*_*jk*_ is the interaction effect between *j*th replication and *k*th block, *B*_*k*_(*G*_*i*_ ) is *i*th genotype in *k*th block, *ER*_*ej*_ is interaction between *e*th environment and *j*th replication, *EB*_*ek*_ is interaction between *e*th environment and *k*th block, *ERB*_*ejk*_ is interaction effect among *e*th environment and *j*th replication and *k*th block, *EB*_*ek*_(*G*_*i*_) is *i*th genotype under interaction of *e*th environment and *k*th block, and *ε_*eijk*_* is the error effect following *N*(0, σ^2^). The broad-sense heritability in multiple environments was showed as following:


$$h^{2}=\frac{\sigma_{B\left(G\right)}^{2}}{\sigma_{B\left(G\right)}^{2}+\frac{\sigma_{EB\left(G\right)}^{2}}{e}+\frac{\sigma^{2}}{er}}$$

where *h*^2^ is the broad sense generalized heritability of average in over multiple environments,$\sigma_{B\left(G\right)}^{2}$is the variance of genotype under block,$\sigma_{EB\left(G\right)}^{2}$is the variance of genotype under environment × block interaction, σ^2^ is the error variance, *e* is the number of environments, and *r* is the number of repetitions in each environment. Significance of each factor was tested by the general linear model method and variance were estimated using mixed method implemented by SAS 9.2 (SAS Institute, Cary, NC, United States).

### SNP Genotyping

DNA samples extracted by CTAB method from RIL6013 were genotyped for SNPs using a soybean SNP660K microarray at Beijing Boao Biotechnology Co., Ltd. A total of 54,836 SNPs were screened on 20 chromosomes. A total of 63,306 SNPs were screened on 20 chromosomes using a soybean SNP180K microarray for SNP genotyping of 455 DNA samples from germplasm resources at Beidahuang Kenfeng Seed Co., Ltd. The obtained SNP markers were screened according to the following criteria: minimum allele frequency for markers (MAF > 5%) and maximum deletion rate <10% for each SNP (Belamkar et al., 2016).

### Bin Maker Map Construction and QTL Localization

Here the SNP data from RIL6013 was used to identify possible crossovers *via* python 2.7snpbinner, and the minimum distance between crossovers is 0.2% of the chromosome length. The aggregated breakpoints generated from the crossover points were then used to create representative bins for the entire population (minimum distance of 30 kb per bin). The obtained bin markers were used to construct a high-density genetic linkage map of SNPbins using the.map function (Linkage map construction) in the software QTL IcimappingV 4.1 (Wang, 2009).

QTL IcimappingV 4.1 (Wang, 2009) software was used to locate additive QTL using two mapping methods: interval mapping (IM-ADD) and inclusive composite interval mapping (ICIM-ADD). The scan step was set to 1.00 cM and the LOD threshold was set to 2.50. The PIN value of the ICIM-ADD method was set to 0.001. The QTL were named using the method of McCouch (1997).

### Genome-Wide Association Analysis

The population structure and LD of the germplasm resource population were described and published earlier by Li X. et al. (2020). The germplasm resource population consisted of two subpopulations containing 132 (29.01%) and 323 (70.99%) lineages, respectively (K = 2). And the physical distance of LD decay was estimated as the position where *r*^2^ dropped to half of its maximum value, the LD decay distance was estimated to be 86 kb.

Genome-wide association analysis was performed using the mrMLM.GUI package (Zhang et al., 2020), and the six methods (mrMLM (Wang et al., 2016), FASTmrMLM (Tamba and Zhang, 2018), FASTmrEMMA (Wen et al., 2019), pLARmEB (Zhang et al., 2017), ISIS EM-BLASSO (Tamba et al., 2017), and pKWmEB (Ren et al., 2018) were used to detect significant QTN. In the first stage, the critical *p*-value parameter was set to 0.005 for all methods except FASTmrEMMA, and the critical LOD value for significant QTN was set to 3 in the final stage. The kinship matrix used in the analysis was also calculated by the software itself.

### Candidate Gene Prediction

Genomic regions repeatedly identified in multiple environments or two populations were used to predict genes involving in PH formation. Specifically, the genome region of QTL interval localized in multiple environments with a genomic region less than 300 kb and the LD decay distance of 86 kb of the QTN localized within the QTL genomic region were selected, and the genes were searched for by the Phytozome website.^2^ The genes expressed in the stems were then screened. Finally, candidate genes related with PH were identified by combining annotation information of genes, pathway analysis in the Kyoto Encyclopedia of Genes and Genomes (KEGG)^3^ and previous studies.

### Candidate Gene Validation

Two parents (Dongnong L13 and Henong 60), two varieties (HN400 and HN451) with lower PH and two varieties (HN369 and HN477) with higher PH, were selected in the RIL6013 population based on the PH phenotype data. The qRT-PCR was used to study the relative expression of candidate genes in these six varieties. These varieties were planted in Harbin in the same environment as E1. Stems were sampled at 10-day intervals starting from the R1 period when elongation is the fastest. The third node down from the top of the main stem was taken and replicated three times per plant. Total RNA was extracted using the OminiPlant RNA Kit (Dnase I) (CWBIO, Jiangsu, China). Two microgram of total RNA was extracted using the EasyScript^®^ One-Step gDNA Removal and cDNA Synthesis SuperMix kit (TransGen Biotech, Beijing, China). The first strand cDNA was synthesized from 2μg of total RNA using the EasyScript^®^ One-Step gDNA Removal and cDNA Synthesis SuperMix kit (TransGen Biotech, Beijing, China). Twenty microliter reaction volume was determined for qRT-PCR using the SYBR^®^Green doping method from Roche Light Cycle™ containing the following components: 10 μL SYBR^®^Green Realtime PCR Master Mix (TOYOBO, Japan), 0.8 μL of each primer (10 μM), 6.4 μL of distilled water and 2 μL of diluted cDNA. The whole reaction was run under the following conditions: pre-denaturation 95°C for 30 s; PCR40 cycles, 95°C for 5 s, 60°C for 20 s, 72°C for 15 s; solubility curve analysis 95°C 10 s, 65°C for 60 s, and 97°C for 1 s. All PCR reactions were repeated three times. Data were processed using the 2^–ΔΔCt^ method using FBOX as the internal reference gene (Bansal et al., 2015), the primers used are shown in Supplementary Table 2.

### Molecular Marker Identification

With the objective to verify the effect of gene and develop markers for molecular assistant selection, the markers with polymorphism in the 100k bp interval of the genes were evaluated for the association with plant height in the 455-germplasm population. The significant differences of averages between allelic genotypes were determined by analysis of variance, and the probability to determine the significance was set 0.05.

## Results

### Phenotypic Variation Analysis

**FIGURE 1:**
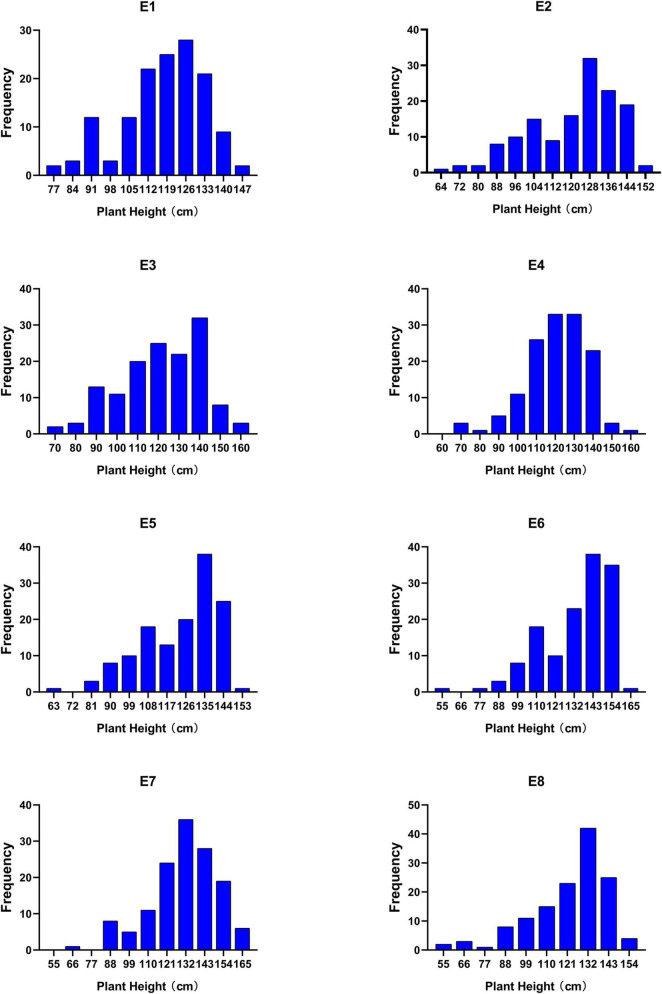
Frequency histograms of plant height in RIL 6013 in eight environments.

Phenotypic data collected from 139 lines of RIL6013 in eight environments were analyzed. 455 germplasm resource populations in four environments were analyzed early by Wang et al. (2021). The results of descriptive statistics (Table 1) showed that the absolute values of kurtosis and skewness were less than 1 in all the eight environments of RIL6013 except E8, which was close to 1. It showed that PH distributed normally (Figure 1). The range of PH in RIL6013 contained those of parents, which indicated a transgressive segregation in the two populations. The coefficient of variation ranged from 13.10 to 22.00% for the RIL6013 population and from 18.03 to 20.31% for the 455-germplasm population, which suggested that a wide range of variation in plant height in two populations and a different genetic basis in different environments.

**TABLE 1:** Descriptive statistics for soybean plant height of RIL6013 population in eight environments and 455 germplasm resources in four environments.

Env.	P_1_^a^	P_2_	Minimum (cm)	Maximum (cm)	Range^b^ (cm)	Mean ± STD^c^ (cm)	Skew	Kurt	CV^d^ (%)
E1	–	–	77.00	144.00	67.00	117.52 ± 15.40	–0.63	–0.22	13.10
E2	–	–	67.40	151.40	84.00	120.49 ± 19.02	–0.75	–0.26	15.79
E3	134.00	63.80	72.00	157.00	85.00	120.96 ± 19.31	–0.48	–0.54	15.96
E4	132.00	63.00	65.00	155.00	90.00	120.42 ± 16.35	–0.83	0.96	13.58
E5	139.67	75.67	65.33	149.00	83.67	123.58 ± 17.97	–0.80	–0.17	14.54
E6	135.00	72.00	60.00	163.33	103.33	133.23 ± 19.47	–0.93	0.68	14.61
E7	144.67	77.50	62.00	162.67	100.67	130.80 ± 19.01	–0.80	0.63	14.53
E8	137.50	77.00	58.00	152.00	94.00	122.04 ± 19.85	–1.20	1.23	16.27
E9	–	–	37.00	131.00	94.00	86.82 ± 15.65	–0.19	0.54	18.03
E10	–	–	38.33	176.67	138.34	95.83 ± 17.44	0.48	2.40	18.20
E11	–	–	40.00	168.67	128.67	97.35 ± 19.77	–0.10	0.08	20.31
E12	–	–	47.00	158.00	111.00	93.13 ± 16.50	0.12	0.22	17.71

*^a^Parents: P1, female cultivar “Dongnong L13”; P2, male cultivar “Henong 60”. ^b^Range, difference between maximum and minimum value. ^c^Mean ± standard deviation of the observed values. ^d^Coefficient of variation.*

The results of ANOVA (Table 2) showed that there were highly significant differences in environment, genotype, and genotype × environment interaction effect, which indicated that PH was influenced not only by genotype and environment but also by genotype by environment interaction effect. Higher broad sense heritability (65 and 72%) was found in RIL6013 and 455 germplasm resource populations, respectively, which indicated that the variation of soybean plant height mainly come from genetic effect.

**TABLE 2:** Joint ANOVA of PH of RIL6013 population and 455 germplasm resources in multiple environment and heritablity.

Population	Source	DF	SS	MS	F	Pr > F	σ^2^
RIL6013	Replication	2	208.033	104.012	0.36	0.6949	
	Environment	7	93199.500	13314.212	46.6	<0.0001	
	Block	9	23934.861	2659.434	9.31	<0.0001	
	Replication × Block	18	8740.392	485.583	1.7	0.033	
	Block (Genotype)	129	351127.234	2721.923	9.53	<0.0001	74.412
	Replication × Environment	14	3107.823	221.994	0.78	0.6954	
	Environment × Block	63	67999.444	1079.362	3.78	<0.0001	
	Replication × Environment × Block	126	38642.481	306.691	1.07	0.2779	
	Environment × Block (Genotype)	896	849663.510	948.291	3.32	<0.0001	220.863
	Error	2050	585738.432	285.733			285.733
	Total	3314	2019279.831				
	h^2^						65%
Germplasm	Replication	2	1353.231	676.612	2.46	0.0852	
	Environment	3	101153.852	33717.952	122.83	<0.0001	
	Block	30	197653.374	6588.451	24	<0.0001	
	Replication × Block	60	17256.132	287.630	1.05	0.3769	
	Block (Genotype)	424	914880.761	2157.744	7.86	<0.0001	139.413
	Replication × Environment	6	381.323	63.553	0.23	0.9665	
	Environment × Block	86	112773.772	1311.321	4.78	<0.0001	
	Replication × Environment × Block	172	45913.361	266.942	0.97	0.5865	
	Environment × Block (Genotype)	1178	753903.492	639.993	2.33	<0.0001	125.211
	Error	3204	879537.283	274.514			274.512
	Total	5165	3071626.461				
	h^2^						72%

### Bin Map and QTL Localization for RIL6013

**FIGURE 2:**
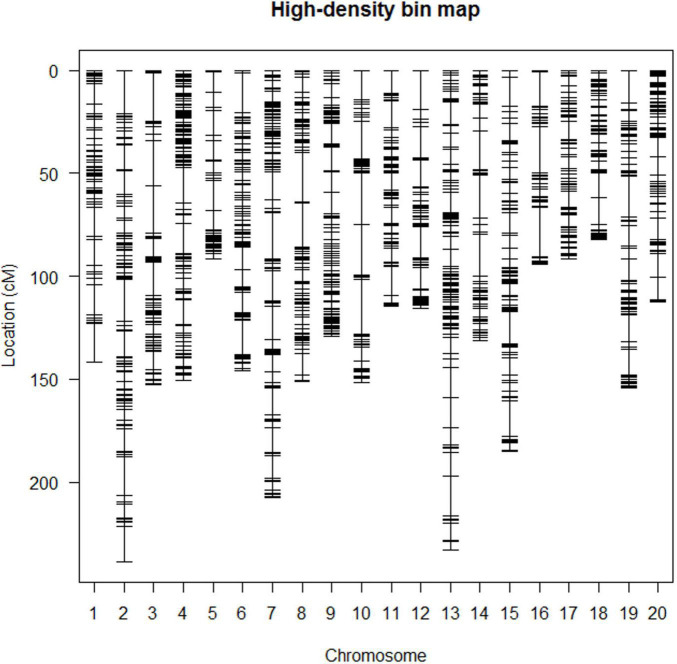
The high-density bin map of RIL6013.

A high-density SNP bin genetic linkage map covered all 20 chromosomes containing 1996 bin markers, and the total length of the map was 2874.72 cM. The number of SNP bin markers per chromosome ranged from 59 to 158, and the length of each linkage group ranged from 82.37 to 238.98 cM. The average number of markers per linkage group was 99.8, and the average distance between markers was 1.48 cM (Figure 2 and Table 3).

**TABLE 3:** Description of characteristics of the 20 linkage groups in the high-density map.

Chr.	No.of SNP in map	Map length (cM)	Average interval (cM)
Chr1	74	141.72	1.94
Chr2	118	238.98	2.04
Chr3	93	152.62	1.66
Chr4	131	150.46	1.16
Chr5	62	91.62	1.50
Chr6	120	145.88	1.23
Chr7	135	207.45	1.55
Chr8	97	150.88	1.57
Chr9	128	129.44	1.02
Chr10	75	151.67	2.05
Chr11	75	114.37	1.55
Chr12	59	115.62	1.99
Chr13	158	232.85	1.48
Chr14	80	131.03	1.66
Chr15	135	184.95	1.38
Chr16	83	94.02	1.15
Chr17	96	91.75	0.97
Chr18	89	82.37	0.94
Chr19	94	154.33	1.66
Chr20	94	112.71	1.21
Sum	1996	2874.72	1.48

**FIGURE 3:**
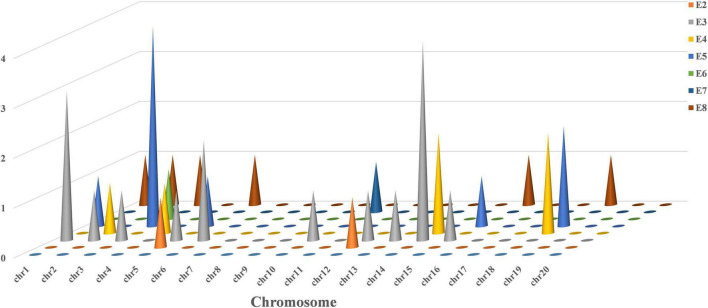
Frequency of QTL for plant height on 20 chromosomes in RIL 6013.

A total of 33 QTLs associated with plant height were localized in the RIL6013 population on 12 chromosomes of soybean using two methods IM and ICIM based on bin mapping (Figure 3 and Supplementary Table 3). The number of QTL localized on each chromosome ranged from one (Chr02, Chr04, Chr12, and Chr13) to six (Chr14), with phenotypic contributions ratio ranging from 0.55 to 13.64%. 2, 16, 6, 9, 1, 1, and 6 QTLs were localized in E2–E8, respectively. A total of three QTL (*qPH-1-1*, *qPH-6-2*, and *qPH-18-4*) showed phenotypic contributions ratio more than 10% and can be considered as the main effective QTL for plant height.

A total of five QTLs were localized in multiple environments (Table 4), and the additive effects were all positive, indicating that the parent Dongnong L13 could increase plant height *via* these QTL. *qPH-2-1* was localized on chr02 in E3, E4, and E8 environments with LOD values of 2.87–3.09 and phenotypic contributions ratio of 1.60–6.46%. The genomic region of *qPH-2-1* was shorter than 320kb, which is suitable for searching candidate genes.

**TABLE 4:** Five QTL detected in multiple environments.

QTL	Env.	Chr.	Marker Interval	LOD^a^	PVE (%)^b^	ADD^c^	Physical Region (Mb)	Method
qPH-1-1	E3/E5/E8/E8	1	60c01056-60c01052	3.34/4.28/3.62/3.62	1.67/3.19/2.68/11.72	16.70/41.16/31.10/31.10	48.83–50.55	IM/IM/IM/ICIM
qPH-2-1	E3/E4/E8	2	60c02058-60c02059	2.87/3.09/2.96	1.60/6.46/3.53	15.53/18.93/27.09	13.66–13.98	IM/IM/IM
qPH-3-5	E5/E6/E8/E6	3	60c03076-60c03080	2.63/2.73/3.51/2.73	4.72/3.92/2.19/3.92	21.31/25.74/32.81/25.74	42.10–42.72	IM/IM/IM/ICIM
qPH-12-1	E2/E3/E2	12	60c12024-60c12032	2.68/2.90/2.68	5.60/1.47/5.60	15.77/15.54/15.77	11.79–14.97	IM/IM/ICIM
qPH-18-3	E5/E8	18	60c18061-60c18058	4.37/3.48	3.25/0.86	24.58/40.77	49.04–52.21	IM/IM

*^a^LOD, logarithm of odds. ^b^PVE, phenotypic variation explained by QTL. ^c^ADD, additive effect.*

### Multi-Locus GWAS for Germplasm

A total of 62 QTN were detected on 18 chromosomes (except for chr11 and chr20) using six multilocus methods within the mrMLM package: mrMLM, FASTmrMLM, FASTmrEMMA, pLARmEB, ISIS EM-BLASSO, and pKWmEB, respectively. LOD values ranged from 3.02 to 10.45, and the ratio of phenotypic variation explained by QTN ranged from 1.12 to 13.12%. Six methods detected 20, 10, 3, 18, 25, and 29 QTN, respectively, while 15, 23, 13, and 11 QTN were detected within E1, E2, E3, and E4, respectively.

Of all QTN, 26 detected by multiple methods were located on chromosomes 1, 2, 3, 4, 6, 7, 9, 10, 13, 14, 15, 16, and 18, respectively, with LOD values ranging from 3.04 to 10.45. The proportion of phenotypic variation explained by QTN ranged from 1.12 to 6.62%. The detected QTN effects (positive or negative) were consistent between methods (Wang et al., 2021).

### Co-detected Regions by Linkage Analysis and Association Analysis

**FIGURE 4:**
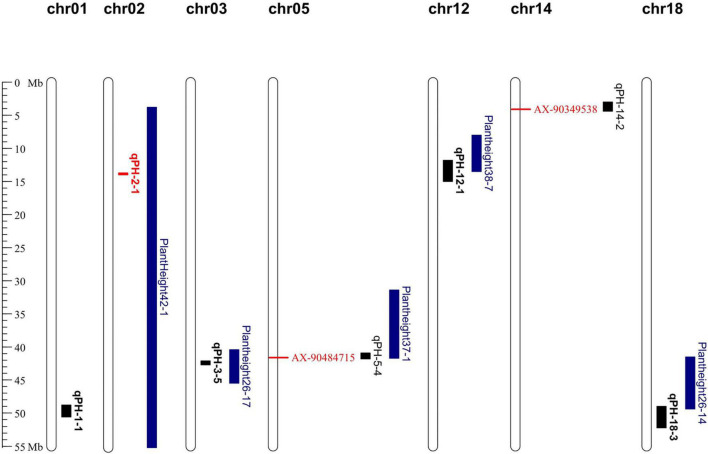
Distribution of QTLs and QTNs for plant height identified in RIL 6013 and the germplasm panel on genome map. Bolded black fonts represent multi-environment QTL, blue fonts represent QTLs in previous studies and red fonts represent QTLs and QTNs that are used to predict candidate genes.

The regions detected by GWAS were compared with those of the linkage analysis. The results showed that two QTN loci fell within the genomic region where the two QTLs identified in the RIL6013 population (Figure 4). Among them, AX-90484715 was located within the interval of *qPH-5-4* and AX-90349538 was located within the interval of *qPH-14-2*. Candidate genes were searched within 43 kb flanking these two QTN loci based on LD distance.

### Candidate Gene Prediction

Based on the above results, candidate genes were selected to search within 13.66–13.98 Mb on Chr02, 41.55-41.63Mb on Chr05 and 4.01-4.09Mb on chr14. A total of 50 candidate genes were searched, of which 46 genes were expressed in the stems. The pathway analysis on 46 genes showed that a total of 18 genes (39.1%) had annotations (Supplementary Table 4). Based on the annotation information of KEGG and metabolic function information, three potential candidate genes were predicted that may be directly or indirectly related to PH (Table 5).

**TABLE 5:** Detailed information of three candidate genes related to plant height.

QTL or QTN name	Gene name	Chromosome	Position	KO number	Annotation
qPH-2-1	Glyma.02G132200	Chr02	13704118..13708015	K09843	CYP707A; (+)-abscisic acid 8’-hydroxylase [EC:1.14.14.137]
qPH-2-1	Glyma.02G133000	Chr02	13760608..13761881	K13448	CML; calcium-binding protein CML
AX-90484715	Glyma.05G240600	Chr05	41566791..41569485	K22736	VIT; vacuolar iron transporter family protein

### Candidate Gene Validation

**FIGURE 5:**
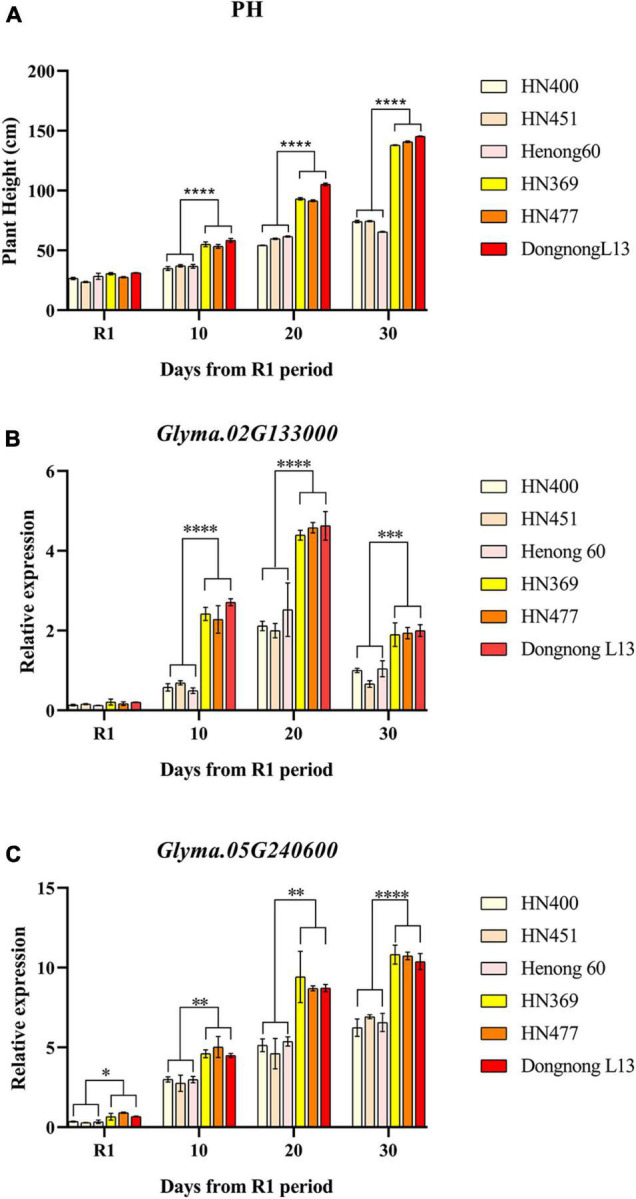
Plant height and relative expression patterns of candidate genes. Plant height of six varieties at different times express as **(A)**, relative expression of Glyma.02G133000 in six varieties at different times express as **(B)** and relative expression of Glyma.02G133000 in six varieties at different times express as **(C)**. *p < 0.05, ^**^p < 0.01, ^***^p < 0.001, ^****^p < 0.0001.

The relative expression of the three candidate genes in the two parents, HN400, HN451, HN369, and HN477, were characterized by applying qRT-PCR. The plant height of the six varieties continued to grow from R1 to day 30, with highly significant differences in plant height from day 10 after R1 (Supplementary Table 5 and Figure 5A). Relative expression amount (REA) of *Glyma. 02G132200* did not differ significantly among varieties at the whole stages, which indicated *Glyma. 02G132200* was not directly related to PH. It could be related to the trait from the DNA level or some other pathway. For *Glyma. 02G133000*, REA of the six varieties increased continuously from R1 to day 20 and started to decrease from day 20 to day 30. REA from day 10 to day 30 of HN369, HN477 and Dongnong L13 was larger significant than that of HN400, HN451 and Henong 60 (Figure 5B). The expression of *Glyma. 05G240600* in the six cultivars continued to increase from R1 to day 30. REA of HN369, HN477 and Dongnong L13 were significantly higher than that of HN400, HN451 and Henong 60 from R1 to day 30 (Figure 5C).

From the 455-germplasm population, seven and one SNP markers associated with plant height were detected near *Glyma.05G240600* and *Glyma.02G133000* (Table 6), which indicated that the two genes controlling plant height. Among these markers, AX-90483488, AX-90490846, and AX-90515514 were detected in three environments, while the rest five markers were detected in only one environment. These eight markers could be used improve plant height commonly or specifically.

**TABLE 6:** SNPs markers associated with plant height near *Glyma.02G133000* and *Glyma.05G240600.1*.

Genes	SNP probe	Physical Position	Genotype	18H^a^		19H		19S	
				F	Pr < F	F	Pr < F	F	Pr < F
*Glyma.02G133000.1*	AX-90394810	13746369	C/T	9.11	0.0027				
	AX-90524676	13750891	A/C	9.01	0.0028				
	AX-90505218	13763345	A/T	4.11	0.0433				
	AX-90351367	13765868	A/G	4.95	0.0267				
	AX-90460406	13766378	A/G			4.12	0.043		
	AX-90483488	13787299	G/C	14.5	0.0002	10.19	0.0015	8.12	0.0046
	AX-90490846	13790192	G/T	7.8	0.0055	7.72	0.0057	10.6	0.0012
*Glyma.05G240600.1*	AX-90515514	41489472	C/T	6.04	0.0144	7.1	0.008	3.93	0.0481

*^a^18H represent the 455-germplasm population was planted in Harbin in 2018, 19H represent Harbin in 2019, 19S represent Shuangyashan in 2019.*

## Discussion

### Improving the Accuracy of QTL Analysis and GWAS by Multi-Environment Experiments and Sufficient SNP Markers

The small amount of RFLP, AFLP, and SSR markers used in previous studies made it difficult to ensure the accuracy of linkage analysis (Singh et al., 2016; Bhat et al., 2020), and most of the previously localized QTL were analyzed in a single environment, which is prone to false positive results (Fang et al., 2020). QTL detected repeatedly in multiple environments are more authentic than those detected in a single environment (Fulton et al., 1997). Here, a high-density genetic map containing 1,966 SNP bin markers was constructed using RIL6013 with the average distance between markers of 1.48 cM, which improved the resolution of the map and facilitated the localization of more QTL and shortened the interval of localized QTL. Phenotypic variation was enriched using an eight-environment experiment at multiple locations over multiple years. And the candidate genes were searched within the stable QTL intervals that were repeatedly localized in multiple environments. Summarizing the above measures, the accuracy of the linkage analysis was improved. For association analysis, more molecular markers could produce a higher probability of detecting functional loci (Xu et al., 2021). Multi-locus GWAS methods are effective in reducing false-positive QTNs compared to single-locus GWAS methods (Qi et al., 2020). An ideal germplasm resource population should contain rich genotypic and phenotypic data (Kaler et al., 2020). Based on the above considerations, the genotype data of 63,306 SNP markers from a natural 455-germplasm population and phenotypic data from four environments were used to conduct multi-locus GWAS analysis, which improve the accuracy of association analysis and reduce the ratio of false positives.

### Comparison With Previous Results of Localized QTL

Here, the five QTLs located by RIL6013 repeatedly in multiple environments and the two QTNs identified by a combination of linkage and association analysis were compared with 238 QTLs associated with PH located by previous researches in the soybase database (Figure 4). The interval of *qPH-2-1*, which is located on chr02, was contained by the interval of Plant height 42-1 (Hu et al., 2013). The interval of *qPH-3-5* on chr03 located in the interval of Plant height 26-17 (Sun et al., 2006). The interval of *qPH-12-1* on chr12 had a overlapping region with the interval of Plant height 38-7 (Lee et al., 2015). The interval of *qPH-18-3* on chr18 crossed the intervals of Plant height 26-13 and Plant height 26-14 (Sun et al., 2006). The genomic region of AX-90484715 on ch05 had a overlapping region with the interval of Plant height 37-1 (Yao et al., 2015). The *qPH-1-1* localized on chr01 in three environments, E3, E5 and E8, was a newly identified QTL, which was more than 20 Mb away from mqPlant height-005 (Pathan et al., 2013). The AX-90349538 on chr14 was a newly identified QTN, which was more than 1.9 Mb away from Plant height 34-6 (Kim et al., 2012). In addition, compared with genes related to plant height identified in previous studies, we found that the *GA20ox* controlling PH formation (Fernandez et al., 2009) was only 170 kb away from AX-90464100. These results support the accuracy of this study.

### Further Analysis of Candidate Genes by qRT-PCR

Using annotation information and metabolic function information we initially predicted three candidate genes that might be associated with PH. Applying qRT-PCR technique to identify the relative expression of the three candidate genes in six varieties with significant differences in plant height, it was found that two candidate genes may be associated with plant height. Among them, *Glyma.02G133000* is a calcium-binding protein gene involved in calmodulin synthesis, and calmodulin is involved in regulating leaf senescence and ABA response in Arabidopsis affecting plant growth and development (Dai et al., 2018). *Glyma.05G240600* is involved in the synthesis of the vesicular iron transporter protein VIT, and iron stored in the vesicles plays a crucial role in the development of plant seedlings; if iron is deficient, it leads to stunted seedlings. Thus, it was speculated to regulate of plant height (Kim et al., 2006). The relative expression of *Glyma.02G133000* and *Glyma.05G240600* from R1 to day 30 was higher in the high PH varieties than in the low PH varieties, so it was speculated that *Glyma.02G133000* and *Glyma.05G240600* may have a positive regulatory function on plant height. However, plant height is a complex quantitative trait, and the specific regulation of plant height by these two candidate genes needs to be investigated in follow-up studies.

## Summary

Here, five multi-environmental QTL and 26 multi-method QTN were detected by linkage analysis and association analysis, respectively, and two candidate genes associated with plant height were identified by pathway analysis and qRT-PCR validation. These results lay the foundation for marker-assisted selection.

## Data Availability Statement

The original contributions presented in the study are included in the article/Supplementary Material, further inquiries can be directed to the corresponding authors.

## Author Contributions

W-XL and HN conceived and designed the experiments. JW, BH, YJ, XH, YG, JC, YL, and JH performed the field experiments. JW and HN analyzed and interpreted the results. JW, BH, and HN drafted the manuscript. W-XL provided the laboratory conditions. All authors contributed to the manuscript revision.

## Conflict of Interest

XH is employed by Key Laboratory of Crop Biotechnology Breeding of the Ministry of Agriculture, Beidahuang Kenfeng Seed Co., Ltd., Harbin, China. The remaining authors declare that the research was conducted in the absence of any commercial or financial relationships that could be construed as a potential conflict of interest.

## Publisher’s Note

All claims expressed in this article are solely those of the authors and do not necessarily represent those of their affiliated organizations, or those of the publisher, the editors and the reviewers. Any product that may be evaluated in this article, or claim that may be made by its manufacturer, is not guaranteed or endorsed by the publisher.

## Footnotes

1
http://soybase.org

2
https://phytozome.jgi.doe.gov

3
https://www.kegg.jp
